# The transcriptome of the entomopathogenic fungus *Culicinomyces clavisporus* contains an ortholog of the insecticidal ribotoxin Hirsutellin

**DOI:** 10.7717/peerj.16259

**Published:** 2023-10-16

**Authors:** Dana Foresman, Aurélien Tartar

**Affiliations:** Department of Biological Sciences, Nova Southeastern University, Fort Lauderdale, FL, United States of America

**Keywords:** Entomopathogens, Fungal ribotoxin, Transcriptomics, Mosquito control, Hirsutellin

## Abstract

The entomopathogenic fungus *Culicinomyces clavisporus* is known to infect and kill mosquito larvae and therefore has been seen as a potential biological control agent against disease vector mosquitoes. Whereas most fungal entomopathogens infect hosts by penetrating the external cuticle, *C. clavisporus* initiates infection through ingestion (*per o*s). This unique infection strategy suggests that the *C. clavisporus* genome may be mined for novel pathogenicity factors with potential for vector control. To this end, an Isoseq-based transcriptome analysis was initiated, and resulted in a total of 3,512,145 sequences, with an average length of 1,732 bp. Transcripts assembly and annotation suggested that the *C. clavisporus* transcriptome lacked the cuticle-degrading proteins that have been associated with other entomopathogenic fungi, supporting the *per os* pathogenicity process. Furthermore, mining of the sequence data unexpectedly revealed *C. clavisporus* transcripts homologous to the Hirsutellin toxin. Comparative sequence analyses indicated that the *C. clavisporus* Hirsutellin predicted protein has retained the canonical molecular features that have been associated with the ribotoxic and insecticidal properties of the original toxin isolated from *Hirsutella thompsonii*. The identification of an Hirsutellin ortholog in *C. clavisporus* was supported by phylogenetic analyses demonstrating that *Culicinomyces* and *Hirsutella* were closely related genera in the Ophiocordycipitaceae family. Validation of the mosquitocidal activity of this novel *C. clavisporus* protein has yet to be performed but may help position Hirsutellin orthologs as prime candidates for the development of alternative biocontrol approaches complementing the current toolbox of vector mosquito management strategies.

## Introduction

The fungus *Culicinomyces clavisporus* is an entomopathogen that was originally isolated from laboratory-reared *Anopheles* spp. mosquito larvae, both in North America and Australia, before being found in natural mosquito populations in these two locations (Scholte et al., 2004). In laboratory conditions, *C. clavisporus* can infect a wide range of mosquito larvae, including vector mosquitoes of public health importance such as *Aedes*, *Anopheles* and *Culex* spp. (Boucias & Pendland, 2012). Because of these attributes, *C. clavisporus* has been considered as a potential biocontrol agent against mosquito populations. Earlier studies aimed at assessing fungal infection efficacy have indicated that highly concentrated conidial suspensions of *C. clavisporus* could result in 100% mortality of *Aedes rupestris* larvae in both laboratory and field conditions (Sweeney & Panter, 1977). Comparative analyses performed in the 1980s demonstrated that larvae of *Ae. aegypti* were more susceptible to this fungus than *Anopheles* spp. (Cooper & Sweeney, 1982), and a more recent study (Rodrigues, Luz & Humber, 2017) confirmed that *C. clavisporus* exposure is remarkably lethal for *Ae. aegypti* (vector of dengue, chikungunya and zika fevers). However pioneer findings also highlighted major limitations for the use of this fungus as a control agent, including the facts that control of target populations could not be sustained for more than one generation, and that the fungus could not remain effective when water temperature exceeded 30 °C (Scholte et al., 2004). These drawbacks have prompted a gradual decrease in *C. clavisporus* consideration for mosquito control alternative tools, which today has resulted in modest records in both scientific literature and molecular data public databases.

Despite the current lack of research activities, *C. clavisporus* has remained a feature in most textbooks (Boucias & Pendland, 2012) and reviews (Scholte et al., 2004) focused on entomopathogenic fungi because of its unique infection pathway that was highlighted by early microscopic observations of the insect-fungal interactions. Entomopathogenic fungi primarily infect their host by attaching to and penetrating the insect external cuticle before reaching the nutrient-rich hemocoel where they propagate and elicit insect death (Mannino et al., 2019). In contrast, infection by *C. clavisporus* is initiated when fungal spores are ingested by the mosquito host and attach to the foregut or hindgut lining, where they germinate into penetrating hyphae that reach the hemocoel (Sweeney, 1975). Earlier studies that reported this *per os* mode of infection were further supported by additional analyses where mosquito larvae were challenged with very high concentrations of *C. clavisporus* conidia. Under these conditions, it was observed that fungal conidia may adhere to the host exoskeleton (head, thorax or abdomen) but without initiating disruption or infection, although infection was observed following attachment to anal papillae (Sweeney, 1979). Detailed observations using electron microscopes focused on the host foregut, which was demonstrated to be the most common site of invasion for *C. clavisporus* (Sweeney et al., 1983). Aside from the unique *per os* mode of infection that is more reminiscent of non-fungal entomopathogens such as the bacteria *Bacillus thuringiensis* (Bravo et al., 2011) or the green alga *Helicosporidium parasiticum* (Tartar, 2013), examinations of mosquito infections also revealed that *C. clavisporus* differs from other entomopathogenic fungi in its ability to rapidly kill its mosquito hosts once infection has been initiated. Whereas common entomopathogenic fungi are known for their long pre-lethal incubation period, which is often seen as an impediment of their biopesticide potential and a rationale for the genetic engineering of faster killing strains (Zhao, Lovett & Fang, 2016), host death during *C. clavisporus* infections has been observed to occur early in the pathogenicity process, and sometimes before the mycelia can colonize the entire insect body cavity (Sweeney, 1983). Secretion of potent insecticidal toxins during host gut ingress has been suggested by early studies focused on mosquito larvae infections (Sweeney, 1983), and this hypothesis was recently supported by a report showing that *C. clavisporus* culture filtrates contain metabolites that act as adulticides for *Ae. aegypti* and other mosquitoes (Singh & Prakash, 2012).

The genome sequences of the most common entomopathogenic fungi have been published and they are typically analyzed using the general paradigm that infection begins with the physical and chemical disruption of the host chitinous external cuticle (Butt et al., 2016; Wang, Leger & Wang, 2016). The molecular arsenal used by *C. clavisporus*, and how it relates to that of other entomopathogenic fungi, has never been investigated. Recently, *de novo* transcriptomics approaches have been successfully used for poorly known entomopathogens, such as the mosquito pathogen *Lagenidium giganteum* (Velasquez et al., 2014) and the ant pathogen *Pandora formicae* (Małagocka et al., 2015). These studies highlighted the value of fast and less expensive transcriptome sequencing efforts as preambles to full scale genome sequencing, and resulted in the preliminary characterization of potential virulence factors, Herein, a similar approach is reported for *C. clavisporus*, as an Iso-Seq-based transcriptome analysis was used to provide insights into the molecular basis of fungal-mosquito interactions.

## Materials & Methods

### Fungal culture, transcriptome sequencing and gene annotation

**Figure 1:**
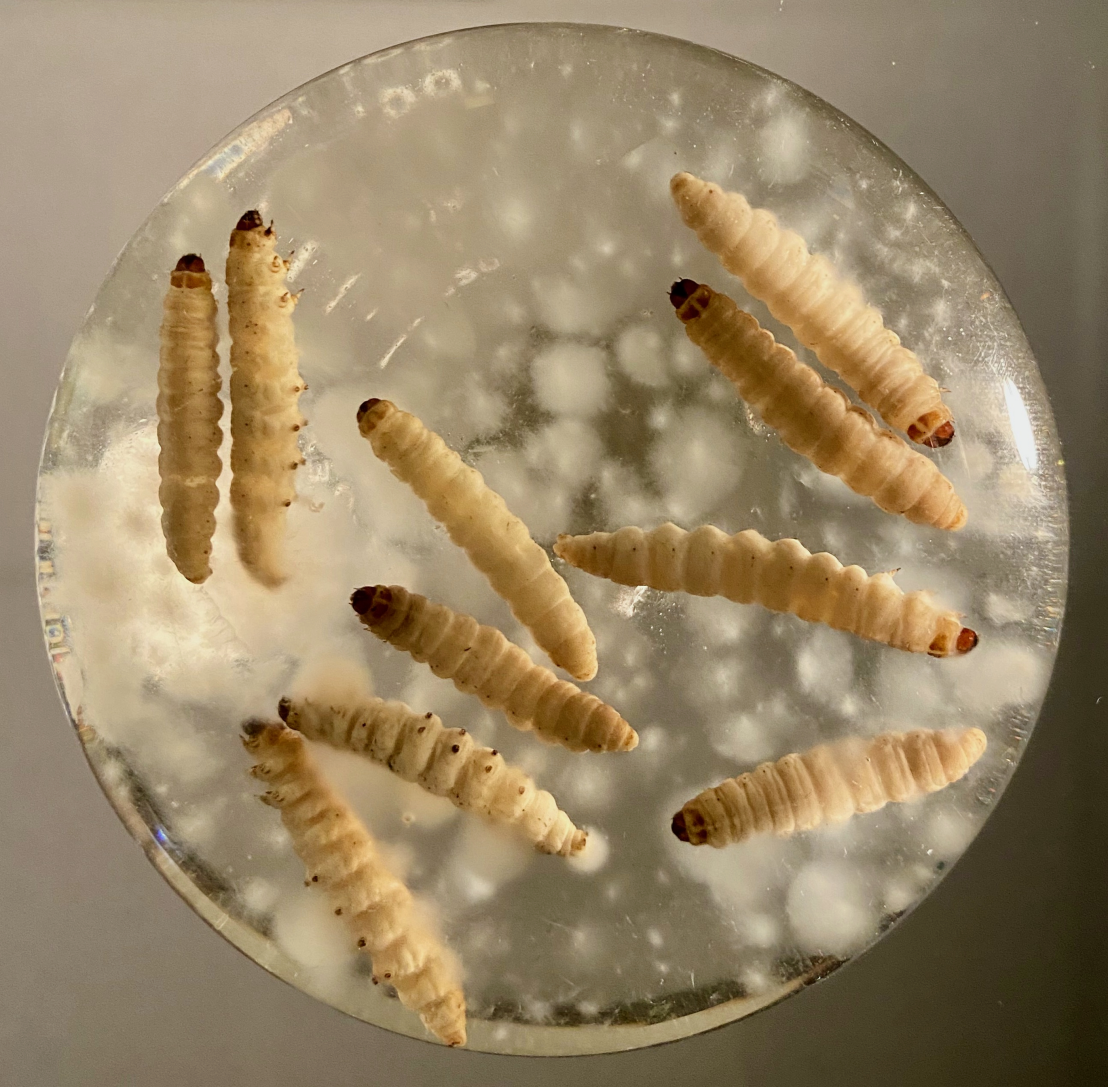
Representative illustration of the growing conditions used to propagate *Culicinomyces clavisporus* prior to RNA extraction. The media was supplemented with whole insect (*Galleria mellonella*) larvae to maximize fungal exposure to insect tissues and elicit the expression of virulence factors.

The fungus *Culicinomyces clavisporus* (ARSEF 582) was obtained from the US Department of Agriculture Agricultural Research Service Collection of Entomopathogenic Fungal Cultures (ARSEF, Ithaca, NY) and grown on plates consisting of agar based Peptone Yeast Extract Glucose (PYG) solid media (Kerwin & Petersen, 1997). Plate cultures were transferred to flasks containing a modified PYG liquid media, where both sources of protein (peptone and yeast extract) were replaced by whole, late instar *Galleria mellonella* larvae (10 larvae per 50 mL of media in 250 mL flasks). The *G. mellonella* larvae were obtained commercially and killed by freezing. Frozen larvae were washed in 95% ethanol, then rinsed in sterile water before being dropped in the media prior to autoclaving, as described previously (Mantilla et al., 2012). Once autoclaved, small agar plugs from *C. clavisporus* PYG plate cultures were used to inoculate the flasks (five replicates), and liquid cultures were left in the dark at 20 °C with no agitation. A two-week incubation period resulted in observable mycelial tissue clusters (Fig. 1). Flask contents (approximately 50 ml) were collected from through filtration. Larvae were removed using sterile tweezers, and mycelial tissue was ground in liquid nitrogen, then processed for RNA extraction using the Qiagen RNeasy Plant Mini Kit. Using the extracted total RNA as starting material, double-stranded cDNA was generated using the LEXOGEN Teloprime Full Length cDNA Amplification kit as per manufacturer’s instructions. Amplified cDNA samples were sent to the University of Florida Interdisciplinary Core for Biotechnology Research (ICBR) where they were assessed for quality and further processed for library construction and SMRT sequencing. Sequencing used the Pacific Biosciences Sequel II system and resulted in the generation and parsing of high quality (“HiFi”, or >Q20) reads. The reads were clustered using CD-hit-est (Huang et al., 2010) using a similarity threshold of 80%. The resulting sequence dataset was annotated in BLAST2GO (Götz et al., 2008) by running BLASTX analyses with an *E* value cutoff of 10^−3^. Transcripts were annotated *in silico* based on these homology searches but function predictions of select sequences, including Carbohydrate-Active Enzymes (CAZymes), were further confirmed by individual Interproscan protein domain searches and signal peptide identification using SignalP version 6 (Teufel et al., 2022).

### Hirsutellin ortholog sequence characterization

The gene-specific primers 5′-GCGGGTCGAGATTGTCAACTGC-3′ (forward) and 5′-CATGACGCCGCAGAACTGAACG-3′ (reverse) were designed from selected sequence reads (*N* = 2) identified as Hirsutellin orthologs. Initially, these primers were used to re-amplify gene fragments *via* internal PCR reactions, but they were also incorporated in RACE (Rapid Amplification of cDNA Ends) PCRs that included cDNA templates obtained by processing the extracted RNA through the SMARTer RACE cDNA amplification kit (Clontech). All amplification reactions were run using the Platinum SuperFi II DNA Polymerase kit (Thermo Fisher, Waltham, MA, USA), and adhered to the manufacturer’s recommendations for a universal annealing temperature (Tm) of 60 °C. Internal and RACE PCR fragments were purified and sequenced commercially using Sanger technologies. Following the generation of a full-length transcript, the predicted protein sequence was annotated through homology (BLAST) and motif (InterProScan) searches. The presence of a signal peptide was assessed using SignalP version 6 (Teufel et al., 2022). The protein sequence was also aligned with various fungal ribotoxin orthologs using MUSCLE (Edgar, 2004). These sequences were accessed from GenBank and included the Hirsutellin A protein sequences from *Hirsutella thompsonii* (accession number AAB47280) and *H. rhossiliensis* (XP_044723119), the anisoplin protein sequences from *Metarhizium brunneum* (XP_014542510) and *M. guizhouense* (KID81433), the alpha sarcin sequence from *Aspergillus giganteus* (BAA02863), the restrictocin sequence from *A. restrictus* (CAA39637), the RNase T1 from *A. oryzae* (P00651) and the newly characterized ageritin protein sequence from *Cyclocybe aegerita* (C0HLG3). Alignment analyses were performed using MEGA X (Kumar et al., 2018) and were complemented by alignment-independent analyses using CLUSS2 (Kelil, Wang & Brzezinski, 2008). Clustering trees were visualized using iTOL (Letunic & Bork, 2021).

### Actin gene amplification, sequencing and phylogenetic analyses

Independent *C. clavisporus* liquid cultures were produced in SDY and were processed for genomic DNA extraction using the Qiagen DNeasy Plant minikit. These DNA preparations were used as templates for PCR amplifications using universal actin primers ED35 (5′-CACGGYATYGTBACCAACTGGG -3′) and ED30 (5′- CTAGAAGCATTTGCGGTGGAC -3′). All PCRs were performed using the following pattern repeated for 30 cycles: 95 °C for 30 s, 50 °C for 30 s, and 72 °C for 1 min. Products were visualized on a 1% agarose gel, extracted, and sequenced commercially (Macrogen, Rockville, MD, USA). Following fragment sequence analysis, gene-specific primers were designed and used to amplify the corresponding cDNA sequences using the RACE cDNA templates described above. Internal and RACE PCR fragments were obtained using the same strategy highlighted for the Hirsutellin gene (above) and led to the generation of a full-length transcript sequence corresponding to the *C. clavisporus* actin gene. The sequence served as input for homology searches (performed in BLASTN) aimed at downloading fungal orthologs from the GenBank database for phylogeny reconstructions, with a focus on mining whole genome shotgun (wgs) contigs sequences from Ophiocordycipitaceae. The accession numbers corresponding to the sequences that were ultimately selected to construct the final alignments are listed as Supplemental material. Sequences were aligned using MUSCLE (Edgar, 2004) and trimmed manually at the 5′ and 3′ ends to produce a 1,002 nucleotide-long block. The file corresponding to this unambiguously aligned dataset was uploaded in MEGA X (Kumar et al., 2018) for both Maximum Likelihood (ML) and Neighbor Joining (NJ) phylogenetic analyses. ML analyses included calculating the best-fit model for the inputted dataset (identified as GTR+I+G) and using this model and parameters for ML tree reconstructions. The calculations, as well as subsequent ML and NJ bootstrap analyses (1,000 replicates) and tree editing were performed in MEGA X.

## Results

### Transcriptome sequencing overview

A total of 3,512,145 high quality reads were obtained from *C. clavisporus* using the SMRT technologies, with an average length of 1,732 bp. There reads are available in the NCBI Sequence Read Archive data under the accession number SRX20105113 as part of Bioproject PRJNA961661. Clustering using an 80% similarity threshold reduced the dataset to 9,318 non-redundant sequences (average length: 1,801 bp), and homology searches demonstrated that 83% of these sequences could be significantly matched to orthologous fungal sequences and tentatively annotated. In agreement with previous observations, the length of each non-redundant sequences impacted the annotation process, as only 62% (1,383 out of 2,222) of the sequences shorter than 1,000bp resulted in significant orthology matches, whereas 90% (6,362 out 7,097) of the sequences longer than 1,000bp were putatively annotated based on homology searches. This served to validate the SMRT-focused approach and supported previous studies that relied on long read technologies for genome-independent, de novo transcriptomic analyses of pathogenic fungi (Elmore et al., 2020). The annotation process revealed that a large fraction of the transcript sequences corresponded to housekeeping genes that included histones, heat shock and ribosomal proteins (data not shown). In contrast, mining the transcriptome for Carbohydrate-Active Enzymes (Drula et al., 2022) highlighted a modest complement of CAZymes (seven sequences). After accounting for reverse complement sequences, analyses demonstrated that CAZyome sequences included single sequences corresponding to members of the Glycoside Hydrolase (GH) families 18, 35, 47 and 125, as well as Glycosyltransferase (GT) family 2 (Table 1). These reads were matched with orthologous sequences from Ophiocordycipitaceae fungi, including the genera *Hirsutella*, *Purpureocillium* and *Drechmeria* (Table 1). Most of these CAZyme enzymes have never been linked to entomopathogenicity, except for GH18 enzymes, which include chitinases (Table 1). GH18 enzymes have been considered as major facilitators of fungal penetration through the host insect cuticle for the majority of entomopathogenic fungi (Mannino et al., 2019), although chitinases are also used by fungi for routine metabolism of the chitin-based fungal cell wall (Thakur et al., 2023). In the *C. clavisporus* dataset, the presence of only one GH18 sequence, combined with the absence of other cuticle degradation genes identified in better studied entomopathogenic fungi, such as Pr1 subtilisins (Gao et al., 2020), strongly contrasted with previously published transcriptome analyses for most entomopathogenic fungi, which have typically been representative of well-established gene family expansions for both proteases and chitinases used during host ingress (Arnesen et al., 2018). For example, a recent *de novo* transcriptome study for the ant pathogen *Pandora formicae* reported 12 subtilisin-like serine protease unigenes and 22 chitinase transcripts (Małagocka et al., 2015). Although it remains unclear if the *C. clavisporus* GH18 transcript listed in Table 1 plays a role in host cuticle degradation, homology searches demonstrated that this sequence is similar to a gene reported from *Hirsutella rhossiliensis* (GenBank accession number XP_044721453). In agreement with this finding, inspection of *the C. clavisporus* transcriptome sequences also revealed several reads matched to the insecticidal toxin Hirsutellin A, which is characteristic of the genus *Hirsutella* (Herrero-Galán et al., 2013). These reads were used as seeds for RACE-PCR amplification and traditional Sanger sequencing, in order to validate and confirm not only the nucleotide sequence of the transcripts, but also their homology to Hirsutellin A.

**Table 1:** *Culicinomyces clavisporus (Cc)* transcripts matched to Carbohydrate-Active Enzymes (CAZymes). Numbers of reads contained in the Iso-Seq library, and best BLASTx hit species are shown. All putative transcripts were represented by only one read, although a minority of reads were identified twice (indicated by an asterisk) as reverse complement sequences. Most transcripts were matched to orthologous protein sequences from Ophiocordycipitaceae fungi. The small number of *C. clavisporus (Cc)* GH18 unigenes contrasted with data reported (Małagocka et al., 2015) in a comparable *de novo* transcriptome analysis performed for the cuticle-degrading fungi *Pandora formicae* (Pf). nr: not reported.

Identified CAZY domain and putative function in *Cc* reads	*Cc* read length	Number of Cc unigenes (*vs*. *Pf*)	Best BLASTX hit species for *Cc* unigenes
GH18- chitinase	2,464 bp	1^*^ [22]	*Hirsutella rhossiliensis*
GH35- beta galactosidase	1,401 bp	1 [nr]	*Fusarium odoratissimum*
GH47- alpha mannosidase	1,410 bp	1 [nr]	*Purpureocillium lilacinum*
GH125-alpha mannosidase	880 bp	1^*^ [nr]	*Drechmeria coniospora*
GT2- glycosyltransferase	1,802 bp	1 [nr]	*Purpureocillium lilacinum*

**Figure 2:**
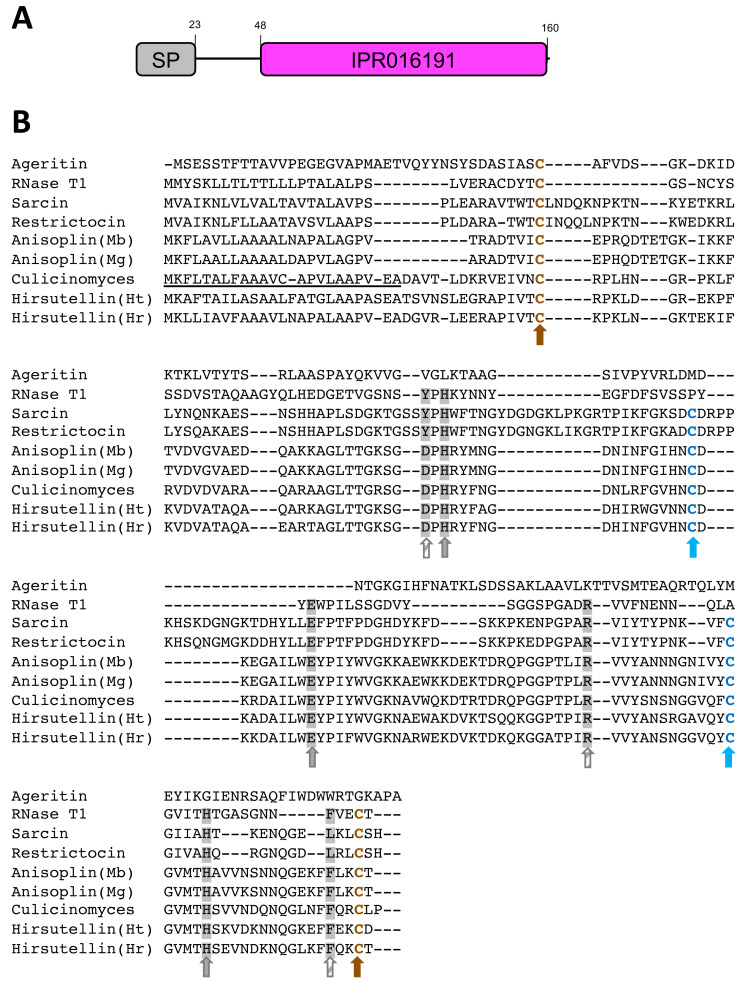
Predicted protein sequence for the *Culicinomyces clavisporus* Hirsutellin ortholog, inferred from full-length cDNA sequence analysis. (A) Schematic illustration of the protein domain organization that combines a short, 23-bp signal peptide (SP, in gray) with a single ribotoxin (IPR016191, in purple) motif. Numbers indicate predicted amino acid positions. (B) Sequence alignment of the C. clavisporus Hirsutellin homolog with previously characterized fungal ribotoxins, including insecticidal toxins from the entomopathogens *Hirsutella thompsonii* (Ht), *H. rhossiliensis* (Hr), *Metarhizium brunneum* (Mb) and *M. guizhouense* (Mg). Cysteine residues are color coded (blue/brown letters and arrows) to highlight their involvement in the formation of the two disulfide bridges associated with the tertiary structure shared by most ribotoxins. The canonical catalytic tryad of His-Ser-His (grey highlights and arrows) is required for ribotoxic activity and is conserved in all ribotoxins except the newly described ageritin. Additional important, but polymorphic, residues are marked with grey highlights but striped arrows. The signal peptide identified in the *C. clavisporus* predicted protein sequence is underlined.

### Hirsutellin A ortholog sequence analysis

**Figure 3:**
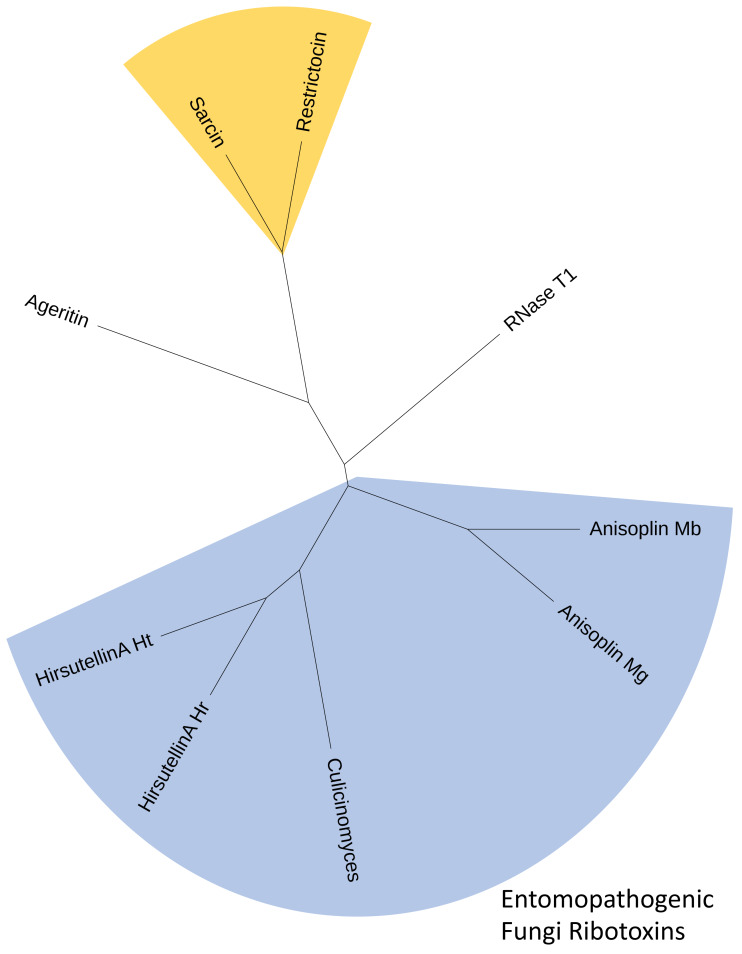
Consensus cladogram depicting clustering of fungal ribotoxins inferred using both alignment-independent methods (CLUSS2) and alignment-based phylogeny reconstruction approaches (UPGMA). The *C. clavisporus* Hirsutellin ortholog appears nested within a clade that include all ribotoxins isolated from entomopathogenic fungi (in blue) and that appears distinct from the *Aspergillus* spp. toxins alpha sarcin and restrictocin (in yellow). All cladograms supported a sister taxa relationship between the sequences from the *Hirsutella* and *Culicinomyces* genera.

RACE PCR reactions resulted in overlapping fragments (5′ end and 3′ end) that were sequenced several times using Sanger technologies and produced high-quality chromatograms. The complete sequence of the Hirsutellin A ortholog generated from the *C. clavisporus* strain ARSEF 582 was 753 bp long, and was deposited in the GenBank/EMBL/DDBJ databases under the accession number OQ873162. *In silico* translation identified a 489 bp Open Reading Frame (ORF) corresponding to a predicted 163 Amino Acid (AA) sequence. As illustrated in Fig. 2A, the domain architecture of the predicted protein consisted of a single ribonuclease motif (IPR016191) preceded by a signal peptide (SignalP likelihood score 99.96%). Homology searches demonstrated that the *C. clavisporus* ribonuclease protein was unique in sequence but most similar to ribotoxin proteins from other well characterized entomopathogenic fungi. The sequence shared 64% identity with the Hirsutellin A protein from *Hirsutella thompsonii* (Maimala et al., 2002), and 60% identity with the recently described anisoplin toxin from *Metarhizium anisopliae* (Olombrada et al., 2017b). Protein sequence alignments inferred from a broader complement of known fungal ribotoxins and RNAses (Fig. 2B) served to further confirm that the *C. clavisporus* ribotoxin orthologous sequence contained the conserved structural features for this family of proteins, including the four cysteine residues involved in the formation of two disulfide bridges, and the amino acids implicated in the active sites. As demonstrated in Fig. 2B, this organization has been found to be characteristic not only of the Hirsutellin A and anisoplin toxins isolated from *Hirsutella* and *Metarhizium* spp., but also of the most representative member of the fungal ribotoxin family: the alpha-sarcin protein from *Aspergillus giganteus*. Studies performed in both alpha sarcin, and its close ortholog, restrictocin, from *Aspergillus restrictus*, have demonstrated that the catalytic tryad consisting of His 50, Glu 96 and His 137 (H-E-H) is required for specific inactivation of ribosomes through interactions with the universally conversed sarcin/ricin loop (SRL) region (Olombrada et al., 2017a). These three amino acids are conserved in most fungal ribonucleases (Fig. 2B), and the more distantly related RNases T1, which are known to contain a distinct disulfide bridge arrangement (Herrero-Galán et al., 2013). In contrast to the *C. clavisporus* sequence, the newly reported insecticidal toxin ageritin (Landi et al., 2019) does not contain either the His-Glu-His motif or the cysteine arrangement to form disulfide bridges (Fig. 2B). In alpha sarcin and restrictocin, other important, but not essential, residues, contribute to the ribotoxin activity: Tyr 48, Arg 121, and Leu 145 (Olombrada et al., 2017a). Although multiple sequence alignments demonstrated that some of these amino acids (Arg) are conserved in Hirsutellin A, some polymorphic sites such as Tyr/Asp (Y/D) have also been identified and assessed for functional implications (Herrero-Galán et al., 2012). As shown in Fig. 2B, the *C. clavisporus* sequence contains the same residues than the Hirsutellin A and anisoplin for these locations: the sarcin Tyr 48 is replaced by Asp, while, in a change similar to the RNase T1 sequence, Leu 145 is replaced by Phe. These features are concordant with the overall sequence alignments (Fig. 2B), and the phylogenetic trees that were inferred from these alignments (not shown), which indicated that the *C. clavisporus* sequence was more similar to the Hirsutellin A and anisoplin proteins than the *Aspergillus* spp. ribonucleases. Both phylogeny reconstructions such as distance-based UPGMA analysis (not shown) and alignment-independent clusterings performed by CLUSS2 (Fig. 3) resulted in similar trees that supported the conclusion that the *C. clavisporus* gene sequence identified in the transcriptome is a Hirsutellin A ortholog that functions as a ribonuclease and is likely to exhibit insecticidal activity. As shown in Fig. 3, all ribotoxins identified in entomopathogenic fungi to date formed a single cluster that was isolated from the *Aspergillus* spp. ribonucleases., and from the highly divergent ageritin. Within the ribotoxin cluster from entomopathogens, the *C. clavisporus* sequence appeared closer related to Hirsutellin proteins than anisoplins (Fig. 3). Because both Hirsutellin A and alpha sarcin have been demonstrated to be lethal when injected in insect larvae (Olombrada et al., 2013), and because these proteins, as well as anisoplins (Olombrada et al., 2017b), have cytotoxic activities when tested against insect cells, the identification of an orthologous sequence in *C. clavisporus* strongly suggests that this fungus produces a ribotoxin protein with potent biological activity against to its insect hosts, and provide a basis to detail the *C. clavisporus* - mosquito host pathosystem at a molecular level.

### Phylogeny reconstructions

**Figure 4:**
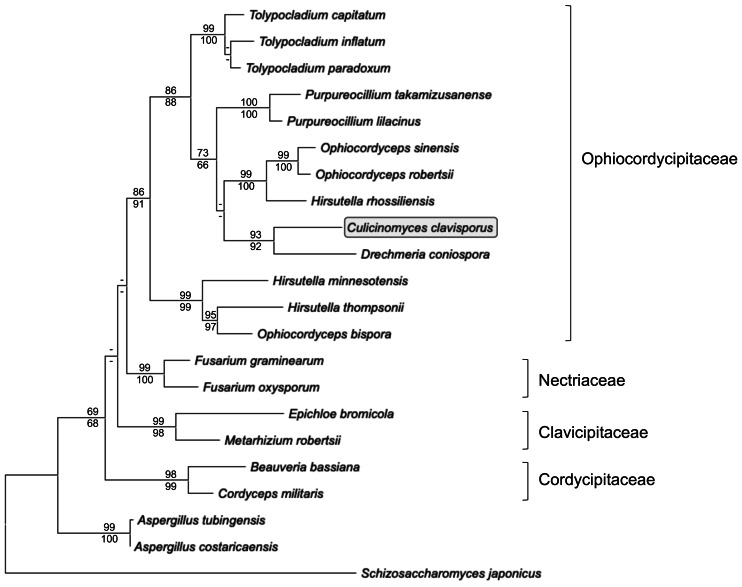
Maximum likelihood phylogram inferred from fungal actin nucleotide sequences (1,002 characters). The tree depicts major monophyletic families within the order Hypocreales (Ophiocordycipitaceae, Clavicipitaceae, Cordycipitaceae and Nectriaceae) and shows the entomopathogen *C. clavisporus* (circled) as a member of the strongly supported Ophiocordycipitaceae family, which contains the Hirsutellin-producing *Hirsutella thompsonii* and *H. rhossiliensis*. Numbers above the nodes represent ML bootstrap values (1,000 replicates), whereas numbers below the nodes correspond to Neighbor Joining (NJ) bootstrap support (1,000 replicates). For clarity, only bootstrap values superior to 60% are indicated (support <60% is represented by -).

Actin gene fragments were amplified and sequenced independently from both gDNA and cDNA preparations obtained from *C. clavisporus*. The gDNA actin fragment (deposited in the GenBank/EMBL/DDBJ databases under the accession number OR335653) was 903-bp long, whereas RACE PCRs resulted in the identification of a full-length transcript that was 1,646-bp long (accession number OR335654). Comparative analyses indicated that the sequences obtained from both sources were identical, but revealed the presence of a small, 55 bp intron in the gDNA fragment at a location that was later shown to be conserved among members of the Ophiocordycipitaceae family (not shown). This intron was omitted from alignments and downstream analyses, and only a portion (1,002 bp) of the actin gene coding sequence was retained to infer phylogenetic analyses and further explore the relationship between the *Culicinomyces* and *Hirsutella* genera. As illustrated in Fig. 4, ML and NJ analyses resulted in trees with very similar topologies: trees were rooted with *Schizosaccharomyces japonicus* and included *Aspergillus* spp. representatives as well as members of four families belonging to the order Hypocreales (Fig. 4). The trees were consistent with previous studies (Quandt et al., 2014; Sung et al., 2007) and depicted the recently proposed Ophiocordycipitaceae family as a strongly supported, monophyletic clade that is distinct from other supported, monophyletic clades corresponding to the Clavicipitaceae, Cordycipitaceae and Nectriaceae families (Fig. 4).The monophyly of the order Hypocreales was also supported, although the nodes indicative of relationships between families within the order were characterized by weaker bootstrap values (Fig. 4). The Ophiocordycipitaceae family clade contained *Hirsutella* spp., and supported a recent proposal to suppress the *Hirsutella* genus name in favor of the synonymous *Ophiocordyceps* (Quandt et al., 2014). However, the monophyly of *Hirsutella*/*Ophiocordyceps* was not resolved (Fig. 4), suggesting that the analyses presented in Fig. 4 may be refined with an increase in taxa and characters sampling. Like *Hirsutella* spp., *C. clavisporus* appeared as a member of the Ophiocordycipitaceae family (Fig. 4), but all phylogenetic trees depicted *C. clavisporus* as sister taxon to *Drechmeria coniospora*, a nematode pathogen (Zhang et al., 2016), rather than with any *Hirsutella* species (Fig. 4). Overall, the phylogenetic relationships inferred using the actin gene sequence (Fig. 4) remained consistent with both the Glycoside Hydrolases homology searches (Table 1), and the analysis of the Hirsutellin sequences (Figs. 2 & 3), demonstrating than *C. clavisporus* is closer related to the genus *Hirsutella* than it is to *Metarhizium* or *Aspergillus*, and belongs to the Ophiocordycipitaceae family.

## Discussion

The main objective of this study was to accelerate gene discovery for *C. clavisporus* and shed lights on the molecular basis of the insect-fungal interactions. The growing conditions (Fig. 1) were designed to mimic previously published strategies (Mantilla et al., 2012; Olivera et al., 2016) aimed at maximizing fungal exposure to insect substrates (including external cuticle) and elicit the expression of virulence factors. However, the selection of a transcriptomics approach, combined with the use of heterologous host larvae (the wax moth *G. mellonella*), carried limitations that may result in an underestimation of the comprehensive array of genes that play a role *in vivo*, in all stages of the infection process. As discussed previously (He, Hu & Xia, 2012), RNA samples collected from liquid-based mycelia cultures (Fig. 1) not only correspond to a single time point but also may only be partially indicative of the gene expression profile used by entomopathogenic fungi during interactions with their hosts. Alternative and complementary approaches, such a complete genome sequencing and time lapsed RNAseq analyses during host infection, are planned for future investigations and may contribute to support the conclusion of this study.

Nevertheless, the transcriptome analysis presented herein represents an entry point for *Culicinomyces* molecular studies and is generally consistent with past microscopic observations of the *Culicinomyces*-mosquito pathosystem. Especially, the absence of genes coding for the main proteins and enzymes associated with cuticle adhesion (adhesins, hydrophobins) and degradation (Pr1 subtilases, chitinases) in other entomopathogenic fungi (Butt et al., 2016) supports findings that demonstrated that the main mode of *C. clavisporus* infection does not involve extensive disruption of the host external cuticle. The mosquito larvae foregut and hindgut regions (as well as anal papillae) are lined with chitin-based cuticle, but these layers have been described as unusually thin compared to the external exoskeleton, and fine observation reports of the initial phase of the *C. clavisporus* infectious cycle remarked that fungal cells easily progressed through these barriers (Sweeney et al., 1983). The small complement of carbohydrate active enzymes (including a single chitinase gene) noted in the transcriptome sequences generated in this study (Table 1) may reflect that both attachment and disruption of the thin layer of host gut cuticle by *C. clavisporus* are achieved with limited chemical breakdown or rely on an array of novel gut-specific proteins that remain to be identified. As demonstrated in this study, instead of the traditional chitin-degrading arsenal, mining of the transcriptome sequence data unexpectedly revealed an unambiguous ortholog to the insecticidal toxin Hirsutellin (Fig. 2), supporting earlier hypotheses associated with notably fast host death occurrences. Complete genome sequencing for *C. clavisporus* will serve to confirm these results, such as a reduced complement of cuticle degrading enzymes typically secreted by entomopathogenic fungi. The full genome sequence may also be searched for additional metabolites that might act in synergy with the toxin reported in this study. Presently, the finding that *C. clavisporus* secretes Hirsutellin orthologous proteins not only significantly impacts the understanding of the phylogenetic affinities of the genus *Culicinomyces*, but also provides a basis to decipher the molecular basis of the mosquitocidal activities that are characteristic of this fungus.

The presence of an Hirsutellin ortholog in the *C. clavisporus* transcriptome (Figs. 2 and 3), combined with the phylogenetic signals inferred from additional analyses described in this study (Table 1, Fig. 4), strongly suggest that *C. clavisporus* is an evolutionary relative of *Hirsutella* spp., which include several pathogens of nematodes, mites and insects (Reddy et al., 2020) in the recently proposed Ophiocordycipitaceae family. Cultures of *H. thompsonii* were the source material for the original discovery of the toxin Hirsutellin (Mazet & Vey, 1995). Because of its activities against mites, *H. thompsonii* was also developed as an acaricidal biopesticide, and remains the first mycopathogen registered in the United States (Boucias & Pendland, 2012). An initial attempt to resolve the phylogenetic placement of *C. clavisporus* was based on actin sequences and confirmed that both *Hirsutella* and *Culicinomyces* shared a common ancestor as members of the Ophiocordycipitaceae family (Fig. 4). However, the unresolved monophyly of the genus *Hirsutella* (Fig. 4) suggested that these preliminary analyses may be refined by comprehensive phylogenomics reconstructions that will use the published genome sequences of *H. thompsonii* (Agrawal et al., 2015), *H. sinensis* (Jin et al., 2020) and *Drechmeria coniospora* (Zhang et al., 2016), among others. These analyses, combined with mining of Ophiocordycipitaceae genomes for additional Hirsutellin orthologs, are currently under way and aimed at investigating a parsimonious shared ancestry for fungal species with similar toxin profiles. A close relationship between the genera *Culicinomyces* and *Hirsutella* has never been suggested but the molecular affinities demonstrated in this study appear concordant with microscopical surveys of the respective pathogenicity processes for these fungi. Because of the mite small size, the mode of infection of *H. thompsonii* has been difficult to clearly establish and has been typically assumed to match the cuticle-degrading paradigm of most entomopathogenic fungi (Reddy et al., 2020). However, detailed microscopic observations of the honeybee/*Varroa* mite pathosystem revealed that *H. thompsonii* conidia did not attach or penetrate the mite host cuticle but initiated the infection process on the membranous arolium of the mite leg suckers (Peng, Zhou & Kaya, 2002). In addition, the same study indicated that *H. thompsonii* conidia did not appear to attach on the bodies of honeybees (Peng, Zhou & Kaya, 2002). The combination of microscopy and molecular information suggests that fungi within the Ophiocordycipitacaeae clade may share a pathogenicity process that avoids relying on exoskeleton drilling but favors (i) targeting less protected locations for host ingress and (ii) producing highly effective insecticidal ribotoxins early in the infection process. Alternative infection pathways for entomopathogenic fungi have been the subject of a recent review (Mannino et al., 2019), and warrant further attention.

Finally, although the activity of the *C. clavisporus* protein reported in this study remains to be tested and validated against host insects, the identification of an Hirsutellin ortholog in a mosquito pathogen is supported by data collected during the initial characterization of this toxin (Mazet & Vey, 1995). In addition to activity against wax moth (*Galleria mellonella*) larvae, bioassays demonstrated that the Hirsutellin protein is lethal when ingested by *Ae. aegypti* larvae (Mazet & Vey, 1995), strongly suggesting that the orthologous protein identified in *C. clavisporus* play a central role during mosquito larvae infection and death. Small concentrations of Hirsutellin caused high mortality (>80%) *per os* in neonate mosquitoes in as little as 24 h, whereas injection bioassays on *G. mellonella* required higher concentrations of Hirsutellin and as high as 15 to 30 days of incubation to cause similar mortality rates (Mazet & Vey, 1995). Based on all these concordant observations, the efficacy of orthologous Hirsutellin proteins on mosquito larvae should be precisely assessed. The threats of widespread resistance to current control tools in *Anopheles* (Ranson & Lissenden, 2016) and *Aedes* (Dusfour et al., 2019) vector populations have alarmed public health experts and stressed the need for alternative strategies. The discovery of an established insecticidal toxin in a pathogen that has been intimately linked (and likely co-evolved) with mosquitoes provides a strong foundation to develop road maps aimed at translating this knowledge into efficient biopesticides. Additionally, the *per o* s mode of action of the Hirsutellin toxin, which has been validated for *Ae. aegytii* larvae (Mazet & Vey, 1995), may act synergically with the toxins isolated from the mosquito biocontrol agent *Bacillus thuringiensis* ser. *israelensis* (Ben-Dov, 2014), contributing to the development of multimodal larvicide strategies. Preliminary work is under way in our laboratory to estimate the impact of Hirsutellin orthologs from both *C. clavisporus* and *H. thompsonii* on *Ae. aegypti* larvae, and use mutagenesis assays to investigate the molecular basis of potential efficacy differences between these two toxins. This on-going work will serve to confirm the functional prediction highlighted in this study and demonstrate that *C. clavisporus* infect and kill mosquitoes in part because it secretes Hirsutellin toxins.

## Conclusions

A large-scale sequencing effort demonstrated that a transcriptome from the fungus *Culicinomyces clavisporus* contains transcripts homologous to the Hirsutellin toxin. Comparative sequence analyses indicated that the *C. clavisporus* Hirsutellin predicted protein has retained the canonical molecular features that have been associated with the ribotoxic and insecticidal properties of the original toxin isolated from *Hirsutella thompsonii*. The identification of an Hirsutellin ortholog in *C. clavisporus*, combined with the preliminary phylogenetic reconstructions that placed the genus *Culicinomyces* within the family Ophiocordycipitaceae, represent a primer for further phylogenomic analyses aimed at resolving the ancestry of *Culicinomyces* and *Hirsutella*. Although the comprehensive functional characterization of the *C. clavisporus* protein expected *per os* mode of action has yet to be performed, Hirsutellin orthologs appear as prime candidates for the development of alternative biocontrol approaches that may complement the current toolbox of vector mosquito management strategies.

## Supplemental Information

10.7717/peerj.16259/supp-1 Supplemental Information 1 Accession numbers (GenBank) for actin sequences used in the phylogenetic analysis

## Additional Information and Declarations

### Data Availability

The following information was supplied regarding data availability: The reads corresponding to the Iso-Seq-based transcriptome analyses described in the study are available in the NCBI Sequence Read Archive: SRX20105113; PRJNA961661. The complete sequence of the Hirsutellin A ortholog generated from the *C. clavisporus* strain ARSEF 582 are available in the GenBank/EMBL/DDBJ databases: OQ873162. The sequences corresponding the actin gene are available at GenBank: OR335653–OR335654.
