# Supplementary material for: The transcriptome of the entomopathogenic fungus Culicinomyces clavisporus contains an ortholog of the insecticidal ribotoxin Hirsutellin
Source: PeerJ. 2023 Oct 16;11:e16259. doi: 10.7717/peerj.16259 (PMC10586291; doi:10.7717/peerj.16259)
Supplement: Supplemental Information 1 [file peerj-11-16259-s001.docx]

| Species name | Family | Accession number |
| --- | --- | --- |
|  |  |  |
| Hirsutella_rhossiliensis | Ophiocordycipitaceae | XM_044861584 |
| Hirsutella_minnesotensis | Ophiocordycipitaceae | JPUM01000388 |
| Hirsutella_thompsonii | Ophiocordycipitaceae | APKB01000030 |
| Ophiocordyceps_sinensis | Ophiocordycipitaceae | NGJJ01000458 |
| Ophiocordyceps_robertsii | Ophiocordycipitaceae | JAPEBW010000961 |
| Ophiocordyceps_bispora | Ophiocordycipitaceae | FKNF01000009 |
| Tolypocladium_paradoxum | Ophiocordycipitaceae | PKSG01000322 |
| Tolypocladium_capitatum | Ophiocordycipitaceae | NRSZ01000823 |
| Tolypocladium_inflatum | Ophiocordycipitaceae | QEPG01000008 |
| Drechmeria_coniospora | Ophiocordycipitaceae | XM_040802987 |
| Purpureocillium_takamizusanense | Ophiocordycipitaceae | XM_047986420 |
| Purpureocillium_lilacinus | Ophiocordycipitaceae | GU299860 |
| Fusarium_graminearum | Nectriaceae | XM_011328784 |
| Fusarium_oxysporum | Nectriaceae | XM_018378442 |
| Schizosaccharomyces_japonicus |  | XM_002174275 |
| Aspergillus_tubingensis | Aspergillaceae | XM_035497066 |
| Aspergillus_costaricaensis | Aspergillaceae | XM_025685792 |
| Beauveria_bassiana | Cordycipitaceae | HQ232398 |
| Cordyceps_militaris | Cordycipitaceae | CP023327 |
| Metarhizium_robertsii | Clavicipitaceae | XM_007825554 |
| Epichloe_bromicola | Clavicipitaceae | CP098269 |
|  |  |  |
